# Complete Genome Sequences of Five Isolated Pseudomonas Strains that Catabolize Pentose Sugars and Aromatic Compounds Obtained from Lignocellulosic Biomass

**DOI:** 10.1128/mra.00987-21

**Published:** 2022-04-04

**Authors:** Mee-Rye Park, Bonnie Fong, Taqwa Tofaha, Blake A. Simmons, Steven Singer

**Affiliations:** a Joint BioEnergy Institute, Emeryville, California, USA; b Biological Systems and Engineering Division, Lawrence Berkeley National Laboratory, Berkeley, California, USA; c University of California, Berkeley, Berkeley, California, USA; Montana State University

## Abstract

We report on complete genome sequences of five Pseudomonas soil isolates that are capable of metabolizing pentose sugars and aromatic monomers. These complete genome sequence data provide insight into possible alternative hosts for the production of biofuels and bio-based chemicals from lignocellulosic feedstock.

## ANNOUNCEMENT

Lignocellulosic biomass from plants is the most abundant and renewable source available for bioconversion (1). Pseudomonas putida KT2440 is a promising host for the production of biofuels and bio-based chemicals, which are currently produced from lignocellulosic hydrolysates (2–5). There has been growing interest in maximizing the range of biomass components to include pentose sugars (e.g., xylose and arabinose), the most abundant components of hemicellulose from grasses (6, 7). However, P. putida KT2440 lacks the native ability to metabolize pentose sugars. While various approaches have been used to utilize pentose sugars through the heterologous expression of pentose sugar pathways in P. putida KT2440 (6–9), several limitations, such as low growth rate, long lag phase, and phenotypic instability, remain.

Here, we report five Pseudomonas isolates recovered from soils from different sites in Emeryville, California, that grow on pentose sugars. Soil samples were inoculated into M9 medium at approximately 2.5% (wt/vol). Serial dilutions were initially plated onto Pseudomonas isolation agar (PIA). Visible colonies were restreaked on M9 minimal media agar plates containing 0.5% (wt/vol) xylose and then on plates with 0.5% (wt/vol) *p*-coumarate as the sole carbon and energy source and were incubated at 30°C. Single bacterial colonies were picked and restreaked on the same medium several times for purification. The growth of each colony was monitored overnight at 30°C in liquid minimal medium (2) supplemented with 0.5% (wt/vol) glucose, xylose, or *p*-coumarate as the sole carbon source. Depending on the growth rate, end optical density at 600 nm (OD_600_), and lag phase, five isolates were finally selected.

**FIG 1:**
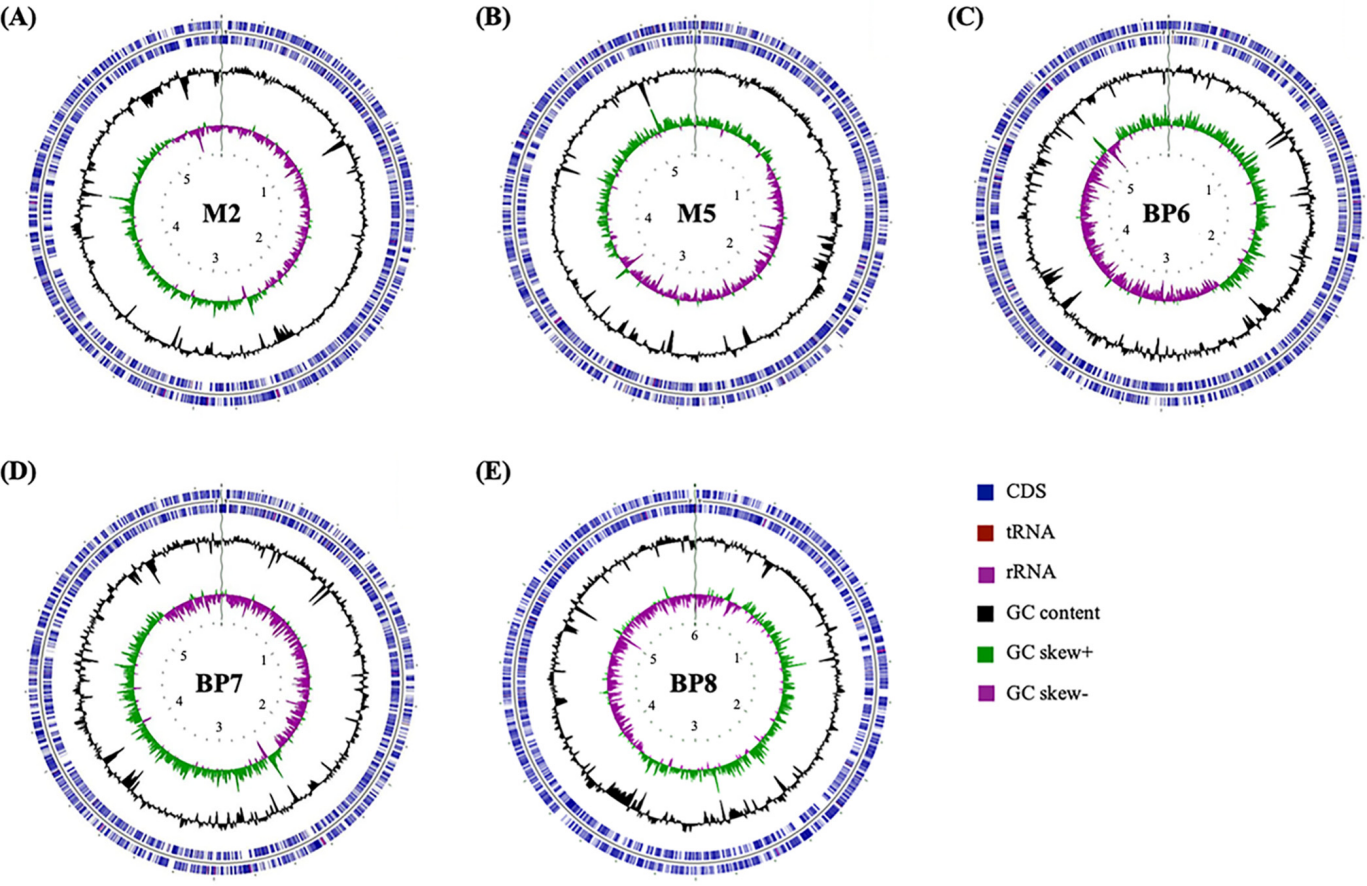
Circular maps representing the genomes of M2 (A), M5 (B), BP6 (C), BP7 (D), and BP8 (E). Forward-strand and reverse-strand CDSs (blue) are depicted on the outermost two circles of the map, and RNA genes (tRNA, red; rRNA, violet) are shown on the same circles. GC content (black) and GC skew (positive GC skew, green; negative GC skew, violet) are drawn on the third and fourth circles, respectively. The scale (in mega-based pairs, mbp) is indicated on the innermost circle. CGView software (19) was used to construct the genome map.

The soil isolates were grown overnight at 30°C in 5 mL LB broth with agitation for the isolation of high-molecular-weight genomic DNA as described previously (10). Pacific Biosciences (PacBio) SMRTbell library preparation (>10 kb, multiplexed) and long-read sequencing using the PacBio Sequel platform (11) were performed by the DOE Joint Genome Institute (JGI). The PacBio reads were filtered to remove reads missing dumbbells on the ends using BBTools (12). Reads of >5 kb were assembled with the Hierarchical Genome Assembly Process (HGAP) v4 (1.0) (smrtlink/8.0.0.80529) (13). Prodigal (14) was used to predict coding sequences (CDSs) on each contig, and the output protein sequences were aligned to the NCBI nonredundant database using DIAMOND (15). Contigs with a probability of being a plasmid were identified using TensorFlow (16). Gene annotations were completed within the JGI Integrated Microbial Genomes (IMG) platform (17) and KBase. Default parameters were used for all software. The sequence details are given in Table 1. Key structural features, including GC content, GC skew, and CDSs, are graphically depicted in Fig. 1.

**TABLE 1:** Genome sequence statistics and characteristics for the five isolates

Pseudomonas isolate	Raw sequencing results				Assembly results			Annotation results		
	No. of >5-kb reads	Mean read length for >5-kb reads (bp)	GC content (%)	Coverage (×)	Genome size (bp)	No. of contigs	No. of plasmids	No. of CDSs	No. of tRNAs	No. of rRNAs
M2	430,129	10,088	61.8	202.1	5,737,635	1	1	5,281	75	22
M5	659,815	10,907	61.9	219.0	5,442,015	1	0	4,903	76	22
BP6	837,793	12,136	61.6	198.2	5,928,556	1	1	5,312	77	22
BP7	763,684	11,060	61.6	202.6	5,979,470	1	1	5,397	77	22
BP8	655,709	10,770	61.8	197.3	6,004,477	1	0	5,341	70	22

The average nucleotide identities (ANIs) based on the whole-genome sequences were calculated using FastANI (18). One set of isolates (Pseudomonas sp. strains M2 and M5) and Pseudomonas sp. strain BP8 showed 85.6% and 84.6% ANI, respectively, to P. putida KT2440, whereas the second set of isolates (Pseudomonas sp. strains BP6 and BP7) showed 96.2% ANI to P. putida KT2440. The genome sequences of the isolates will contribute to the understanding and exploration of metabolic pathways of the main carbon sources derived from lignocellulosic biomass and will facilitate genetic engineering.

### Data availability.

The whole-genome sequences for each of the five Pseudomonas species have been deposited in GenBank under the following accession numbers: Pseudomonas sp. strain BP6, JAGINI000000000; Pseudomonas sp. strain BP7, JAGINJ000000000; Pseudomonas sp. strain BP8, JAGINK00000000; Pseudomonas sp. strain M2, JADOUD010000001; Pseudomonas sp. strain M5, JAFBBH000000000. The SRA accession numbers for the raw reads are as follows: Pseudomonas sp. strain BP6, SRX13609329; Pseudomonas sp. strain BP7, SRX13609331; Pseudomonas sp. strain BP8, SRX13609332; Pseudomonas sp. strain M2, SRX9632768; Pseudomonas sp. strain M5, SRX10105427.
